# Supplementary material for: The genome of a globally invasive passerine, the common myna, Acridotheres tristis
Source: DNA Res. 2024 Feb 16;31(2):dsae005. doi: 10.1093/dnares/dsae005 (PMC10917472; doi:10.1093/dnares/dsae005)
Supplement: dsae005_suppl_Supplementary_Material [file dsae005_suppl_supplementary_material.pdf]

The genome of a globally invasive passerine, the common myna, *Acridotheres tristis*

Katarina C. Stuart[1,2], Rebecca N. Johnson[3], Richard Major[4], Kamolphat Atsawawaranunt[1], Kyle M. Ewart[4,5], Lee A. Rollins[2], Anna W. Santure[1], Annabel Whibley[1]

[1]School of Biological Sciences, University of Auckland, Auckland, Aotearoa/New Zealand
[2]Evolution and Ecology Research Centre, School of Biological, Earth and Environmental Sciences, University of New South Wales, Sydney, Australia
[3]National Museum of Natural History, Smithsonian Institution, Washington, DC
[4]Australian Museum Research Institute, Australian Museum, Sydney, Australia
[5]School of Life and Environmental Sciences, University of Sydney, Sydney, Australia

Supplementary materials

Appendix 1: Sampling, extraction, and sequencing

Extractions

High-molecular weight gDNA was extracted using the QIAGEN PureGene Tissue Extraction protocol for linked-read sequencing, while for ONT sequencing, gDNA was extracted with the Qiagen Genomics Tips and buffer set for the first round of sequencing. Subsequent iterations of ONT sequencing used genomic DNA extracted with the PacBio Nanobind BigDNA animal tissue kit and further purified using the Short Read Eliminator reagent after extraction. Messenger RNA was extracted with the Qiagen RNeasy Plus Universal Mini Kit and Qiagen Oligotex mRNA Mini Kit. Nucleic acid concentration and quality was assessed using a NanoDrop, Qubit and TapeStation.

gDNA and cDNA sequencing

High molecular weight gDNA (1 ug) was prepared for 10x Chromium linked-read sequencing according to the manufacturer's recommended protocols. A 10x GEM library was barcoded using the Chromium Genome Reagent Kits (v2 Chemistry). The library was run on a single lane of a S4 flowcell and sequenced using the Illumina Hiseq X Ten sequencing platform (150 bp paired end reads) at Macrogen, Seoul, Republic of Korea.

Oxford Nanopore Technologies ligation sequencing libraries were prepared with 1.5ug gDNA per library. An initial round of sequencing using the LSK109 library kit, subsequent rounds used the LSK110 and LSK112 releases (Table S2). All sequencing was performed on R9.4.1 flow cells on a MinION. A total of 6 flow cells were used. Libraries were loaded (at ~50fmol) multiple times per flow cell, with nuclease flushes to restore pore functionality between loads.

The cDNA was quantified using an Agilent 2200 TapeStation (Agilent, California, USA) and Nanodrop spectrophotometer (Thermo Fisher Scientific). cDNA from three tissues (liver, heart, testes) was then prepared for sequencing using a TruSeq stranded mRNA library prep kit, and sequenced on a NextSeq 75bp PE high output sequencing run at the Ramaciotti Centre for Genomics (UNSW, Sydney, NSW).

Appendix 2: Manual genome curation

The initial nanopore assembled and polished genome assembly was mapped, using MINIMAP2 v2.24 [1], to the zebra finch (*Taeniopygia guttata)* genome assembly (GCF_003957565.2), as well as a Vertebrate Genomes Project (VGP) common myna genome assembly (GCA_027559615.1). These alignments were visualised as dot plots using DGENIES v1.4.0 [2], and the dot plots manually inspected for signatures of misassemblies (Fig. S1). To investigate these putative misassemblies, we used MINIMAP2 to map reads (raw ONT, and trimmed 10x reads) to the assembly so coverage around potential misassembly points could be investigated. In addition to the raw reads, we aligned the assemblies of the European starling (*Sturnus vulgaris*) genome (GCA_023376015.1) [3] to provide extra context around potential misassembly points. These three tracks were visualised in IGV v2.15.4 [4] and locations of misassembly signatures were examined. Contigs that contained evidence for potential misassembly (for examples, see: Fig. S2) were manually broken using BEDTOOLS V2.30 *getfasta* [5]. We used DIPLOIDOCUS v1.1.1 [6] to check for assembly artefacts (runmode=purgehaplotig) and vector contamination (runmode=vecscreen using the NIH UniVec database). NUMTFINDER v0.5.1 [7] was used to identify the mitochondrial genome and search for misassemblies of it, using the common myna mitochondrial genome as a reference (CM050619.1). Contigs below length of 1,000bp were identified using BBMAP v38.81 [8] and removed. BLOBTOOLS v1.0 [9] was used to assess if any of the assembled contigs may have belonged to contaminants in the raw sequencing data, using BLASTN v2.13.0 [10] (-task megablast) to assemble the database, and the ONT reads mapped to the genome using MINIMAP2. Sequences that were identified as contaminants were removed from the assembly (Fig. S3).

Appendix 3: Z chromosome genomic rearrangement

Prior to use as a reference for synteny scaffolding, the VGP *A. tristis* genome was examined for synteny against the *T. guttata* genome using MINIMAP2 and visualised in DGENIES (Supplementary materials: Fig. S5). The dot plot revealed that approximately 17 Mb section of a *T. guttata* autosomal chromosome was assembled onto the end of the Z chromosome of VGP *T. guttata*. To investigate whether this was a genuine genomic rearrangement in *A. tristis* relative to the zebra finch or a misassembly, we mapped our raw ONT reads to VGP *T. guttata* using MINIMAP2 and visualised in IGV (Supplementary materials: Fig. S5). High ONT read concatenation around the scaffold join site did not support the linking of these two sequences in our assembly. We took a conservative approach and excluded the equivalent region in our assembly from synteny based scaffolding, but during scaffold renaming renamed it based on *T. guttata* synteny.

REFERENCES

1. Li, H. 2018, Minimap2: pairwise alignment for nucleotide sequences. *Bioinformatics*, **34**, 3094–100.
2. Cabanettes, F., and Klopp, C. 2018, D-GENIES: dot plot large genomes in an interactive, efficient and simple way. *PeerJ*, **6**, e4958.
3. Stuart, K. C., Edwards, R. J., Cheng, Y., et al. 2022, Transcript- and annotation-guided genome assembly of the European starling. *Molecular Ecology Resources*, **22**, 3141–60.
4. Robinson, J. T., Thorvaldsdóttir, H., Winckler, W., et al. 2011, Integrative genomics viewer. *Nat Biotechnol*, **29**, 24–6.
5. Quinlan, A. R., and Hall, I. M. 2010, BEDTools: a flexible suite of utilities for comparing genomic features. *Bioinformatics*, **26**, 841–2.
6. Chen, S. H., Rossetto, M., van der Merwe, M., et al. 2022, Chromosome-level de novo genome assembly of Telopea speciosissima (New South Wales waratah) using long-reads, linked-reads and Hi-C. *Molecular Ecology Resources*, **22**, 1836–54.
7. Edwards, R. J., Field, M. A., Ferguson, J. M., et al. 2021, Chromosome-length genome assembly and structural variations of the primal Basenji dog (Canis lupus familiaris) genome. *BMC Genomics*, **22**, 188.
8. Bushnell, B. 2014, *BBMap: A Fast, Accurate, Splice-Aware Aligner*. Retrieved from https://escholarship.org/uc/item/1h3515gn, Lawrence Berkeley National Laboratory.
9. Laetsch, D. R., and Blaxter, M. L. 2017, July 31, BlobTools: Interrogation of genome assemblies. F1000Research, F1000Research.
10. Camacho, C., Coulouris, G., Avagyan, V., et al. 2009, BLAST+: architecture and applications. *BMC Bioinformatics*, **10**, 421.

Table S1 | *Acridotheres tristis* individuals whole genome resequenced. SVC = single nucleotide variant calling, RC = recombination profiling, DP = demography PSMC, DS = demography stairway plot.

Country & Status	Population	Abbreviated ID (internal)	Full name (internal)	SRA Accession	Analysis used
INDIA *Native*	Tamil Nadu (TN)	J753	IND_TN_3695_M0298	SAMN38881494	SVC, RC, DS
		J754	IND_TN_3696_M0299	SAMN38881495	SVC, RC, DS
		J755	IND_TN_3697_M0300	SAMN38881496	SVC, RC, DS
		J756	IND_TN_3699_M0302	SAMN38881497	SVC, RC, DS
		J757	IND_TN_3700_M0303	SAMN38881498	SVC, RC, DS
		J758	IND_TN_3701_M0304	SAMN38881499	SVC, RC, DS
		J759	IND_TN_3710_M0305	SAMN38881500	SVC, RC, DS
		J760	IND_TN_3711_M0306	SAMN38881501	SVC, RC, DS, DP
	Madhya Pradesh (MP)	J761	IND_MP_3751_M0317	SAMN38881502	SVC, RC
		J762	IND_MP_3752_M0318	SAMN38881503	SVC, RC
		J763	IND_MP_3753_M0319	SAMN38881504	SVC, RC
		J764	IND_MP_3754_M0320	SAMN38881505	SVC, RC
		J766	IND_MP_3756_M0322	SAMN38881506	SVC, RC
		J767	IND_MP_3757_M0323	SAMN38881507	SVC, RC
		J768	IND_MP_3758_M0324	SAMN38881508	SVC, RC, DP
	Maharashtra subpop A (MAa)	J745	IND_MA_979_M0213	SAMN38881509	DP
	Maharashtra subpop b (MAb)	J748	IND_MA_3731_M0307	SAMN38881510	DP
Fiji *Invasive*	Fiji (FIJI)	J797	FIJI_923_M0203	SAMN38881511	DP
Australia (AUS) *Invasive*	Melbourne (MEL)	J783	AUS_MEL_12408	SAMN38881512	DP
	Cairns (CAI)	J773	AUS_CAI_11606	SAMN38881513	DP

New Zealand (NZ) *Invasive*	Leigh (LEI)	J718	NZ_LEI-06_M0006	SAMN38881514	DS
		J719	NZ_LEI-07_M0007	SAMN38881515	DS
		J720	NZ_LEI-08_M0008	SAMN38881516	DS
		J721	NZ_LEI-09_M0009	SAMN38881517	DS
		J722	NZ_LEI-10_M0010	SAMN38881518	DS
		J723	NZ_LEI-13_M0013	SAMN38881519	DS
		J724	NZ_LEI-18_M0018	SAMN38881520	DS
		J725	NZ_LEI-19_M0019	SAMN38881521	DS, DP
	Napier (NAP)	J731	NZ_NAP-08_M0158	SAMN38881522	DP
	Great Barrier Island (GBI)	J735	NZ_GBI-13_M0133	SAMN38881523	DP

Table S2 | ONT read batches and corresponding program version numbers used for raw data processing used at ONT assembly steps, assessed using NanoStats.

LSK Chemistry	Flow Cell Type	Guppy nucleotide basecalling config	Methylation basecalling config	Total length of raw reads (Mb)	Total length of filtered reads (Mb)	Average read length (bb)	Mean read quality (Q score)
Ligation (SQK-LSK109)	R9.4.1	dna_r9.4.1_450bps_sup.cfg	dna_r9.4.1_450bps_modbases_5mc_cg_sup.cfg	1,459.18	1,211.15	3.11	15.2
Ligation (SQK-LSK110)	R9.4.1	dna_r9.4.1_450bps_sup.cfg	dna_r9.4.1_450bps_modbases_5mc_cg_sup.cfg	4,531.31	3,561.85	11.22	13.9
Ligation (SQK-LSK112)	R9.4.1	dna_r9.4.1_e8.1_sup.cfg	dna_r9.4.1_e8.1_modbases_5mc_cg_sup.cfg	22,645.27	18,779.21	7.17	16.2

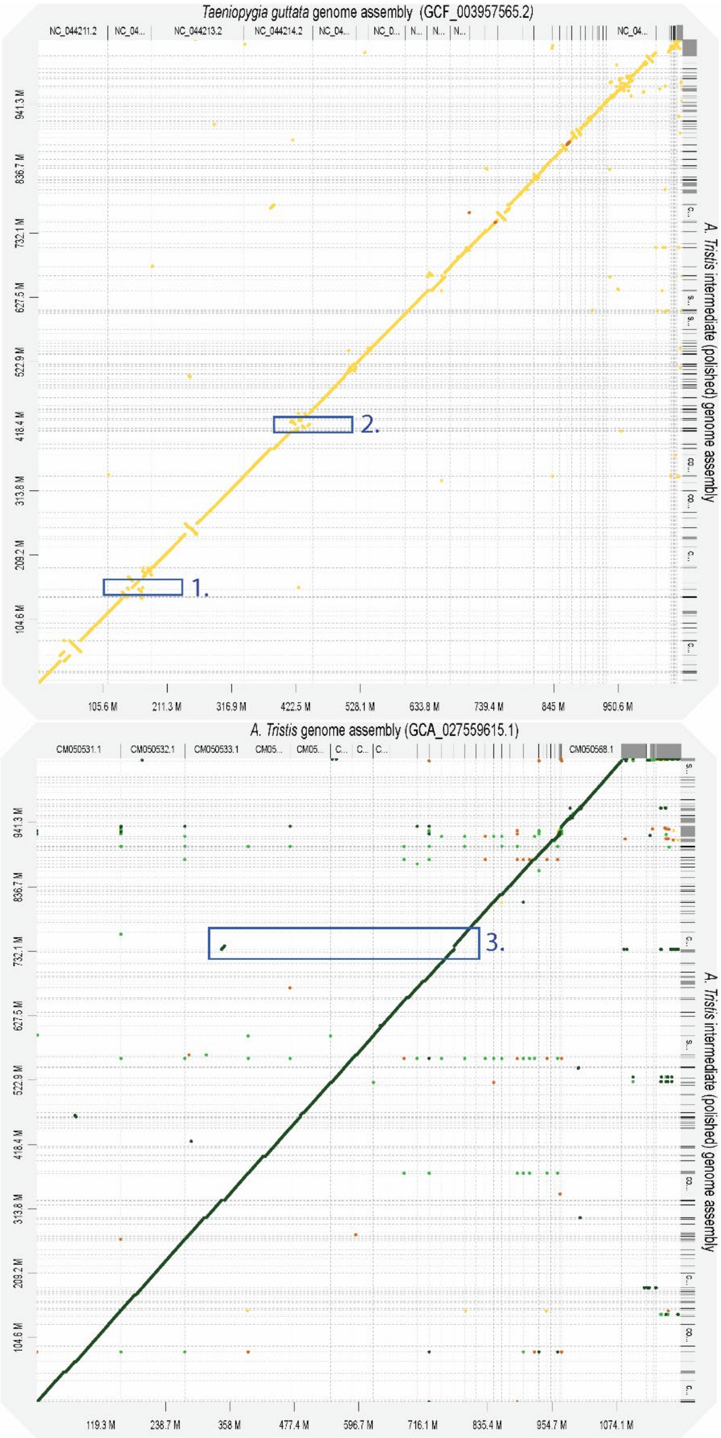

Figure S1 | Dot plot of the polished *Acridotheres tristis* genome (initial genome assembly in this stidy) against the *Taeniopygia guttata* genome (GCF_003957565.2) and VGP *Acridotheres tristis* genome (GCA_027559615.1). Alignments done using MINIMAP2 v2.24 and visualised in DGENIES v1.4.0. Indicated on the dot plot are three exemplar windows that were investigated during manual genome curation that were deemed to be 1) a misassembly, 2) not a misassembly, and 3) a misassembly.

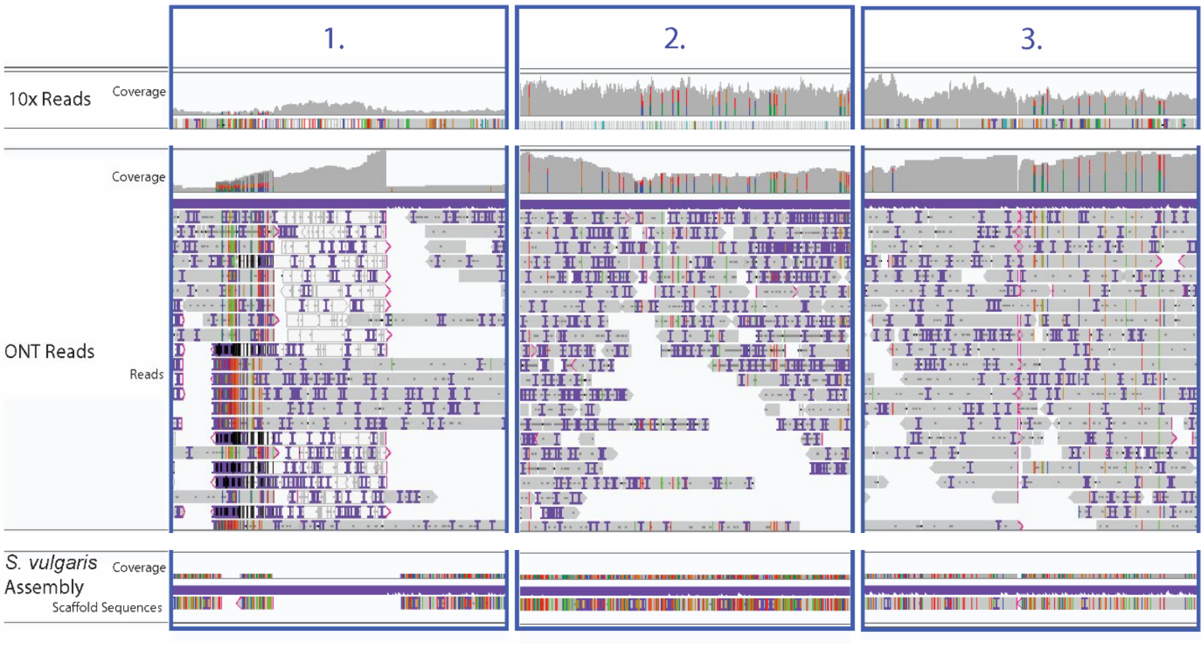

Figure S2 | Examples of manual assembly curation. Alignments produced using MINIMAP2 v2.24 and processed in SAMTOOLS v1.13, and finally visualised in IGV v2.15.4. Window numbers correspond to the exemplar windows from Figure S1 that were investigated during manual genome curation that were deemed to be 1) a misassembly, 2) not a misassembly, and 3) a misassembly.

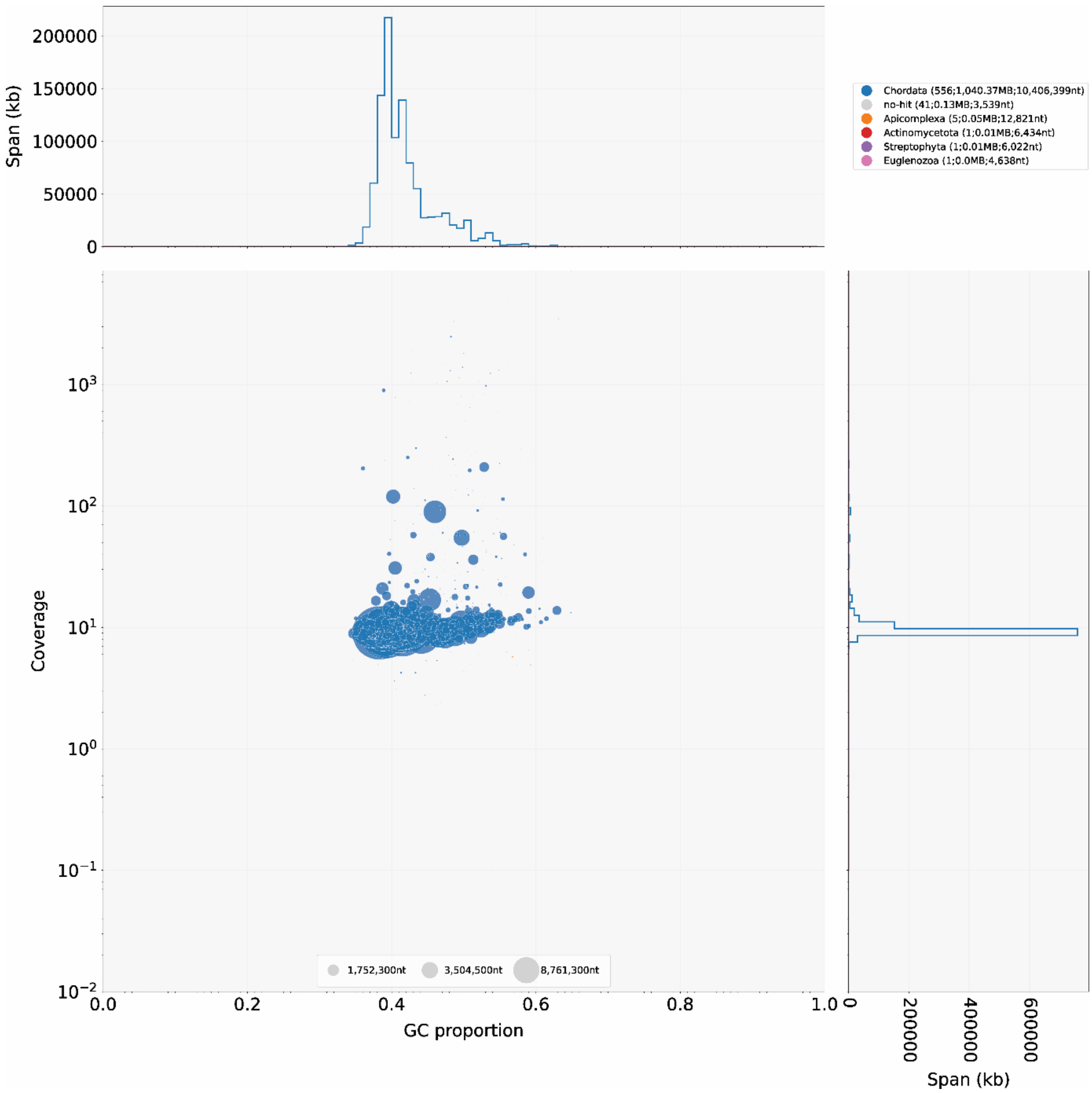

Figure S3 | Blobtools summary plot of genome assembly contamination produced using blast (-task megablast) to assign taxonomic identities to the sequences.

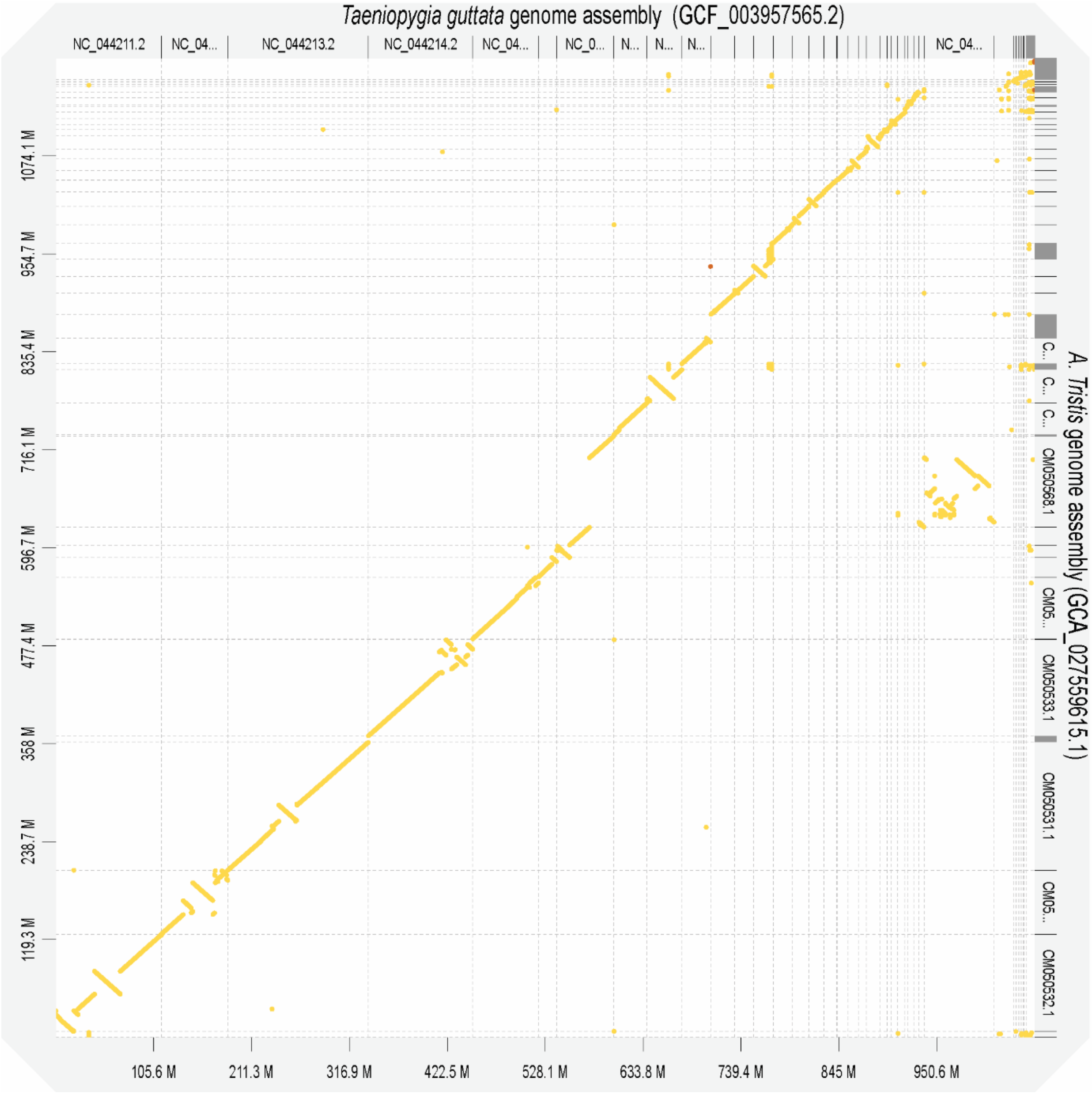

Figure S4 | Dot plot of the VGP *Acridotheres tristis* genome (GCA_027559615.1) and *Taeniopygia guttata* genome (GCF_003957565.2). Alignments done using MINIMAP2 v2.24 and visualised in DGENIES v1.4.0.

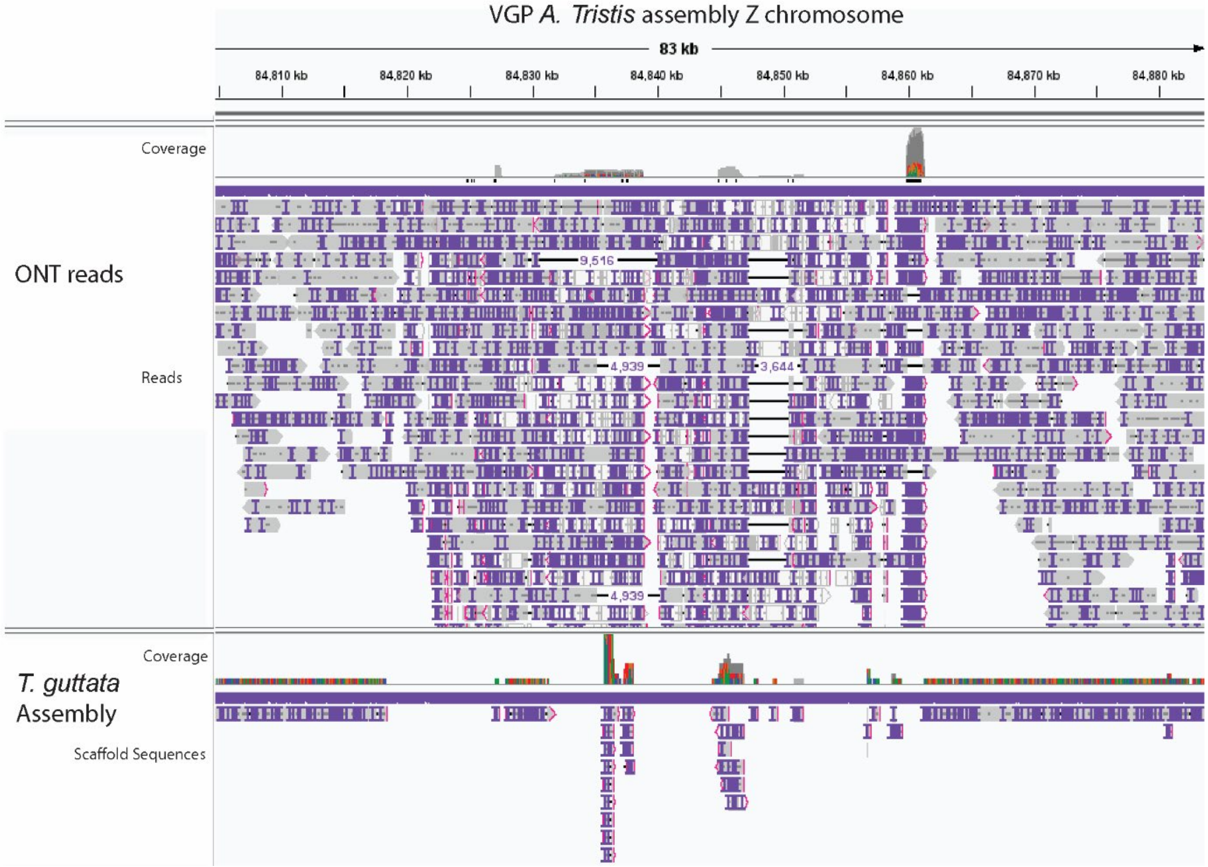

Figure S5 | Manual examination of VGP *Acridotheres tristis* genome (GCA_027559615.1) z chromosome sequence. Alignments produced using MINIMAP2 v2.24 and processed in SAMTOOLS v1.13, and finally visualised in IGV v2.15.4.

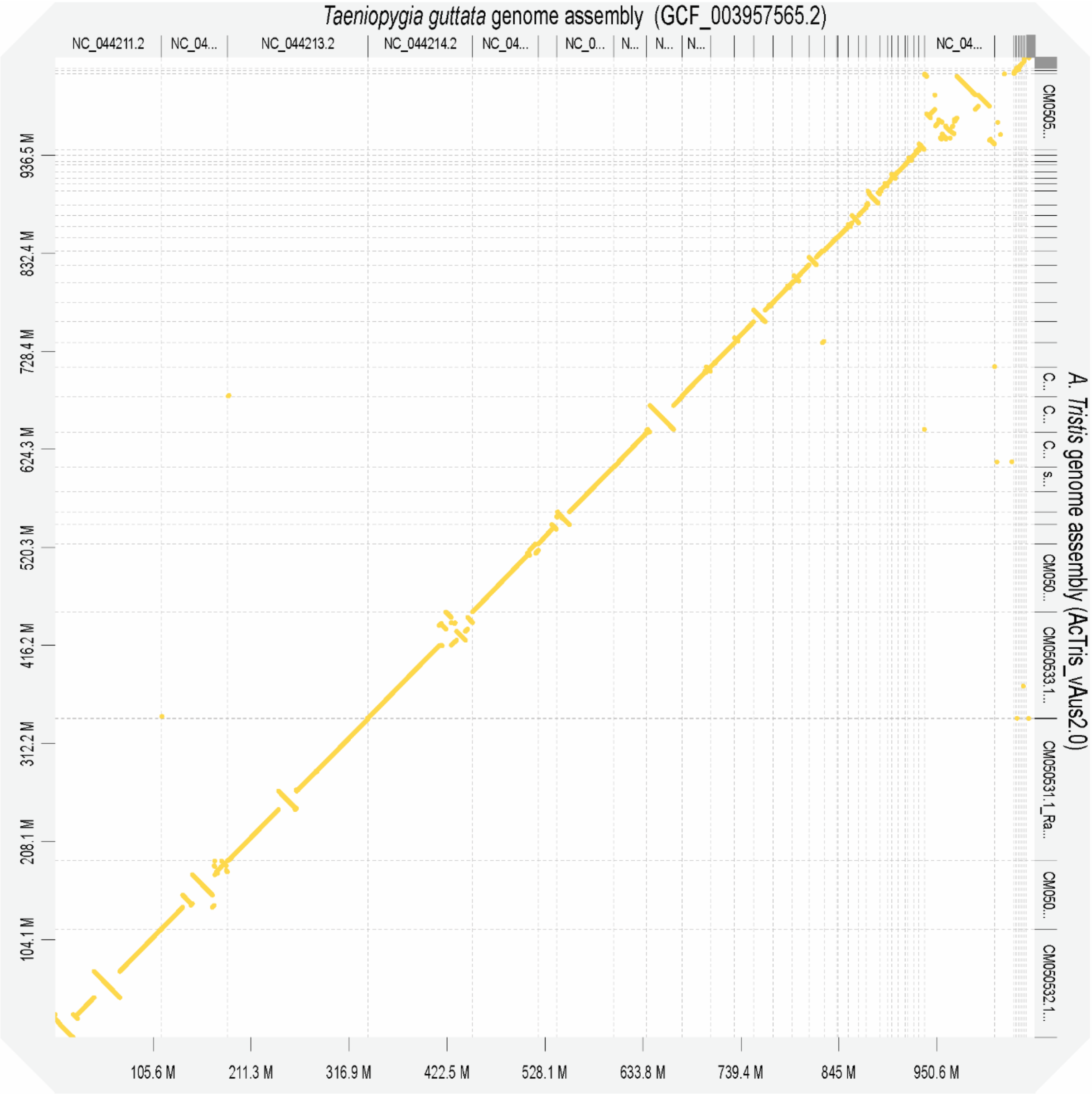

Figure S6 | Dot plot of the *A. trisits* genome assembly (AcTris_vAus2.0) and *Taeniopygia guttata* genome (GCF_003957565.2). Alignments done using MINIMAP2 v2.24 and visualised in DGENIES v1.4.0.

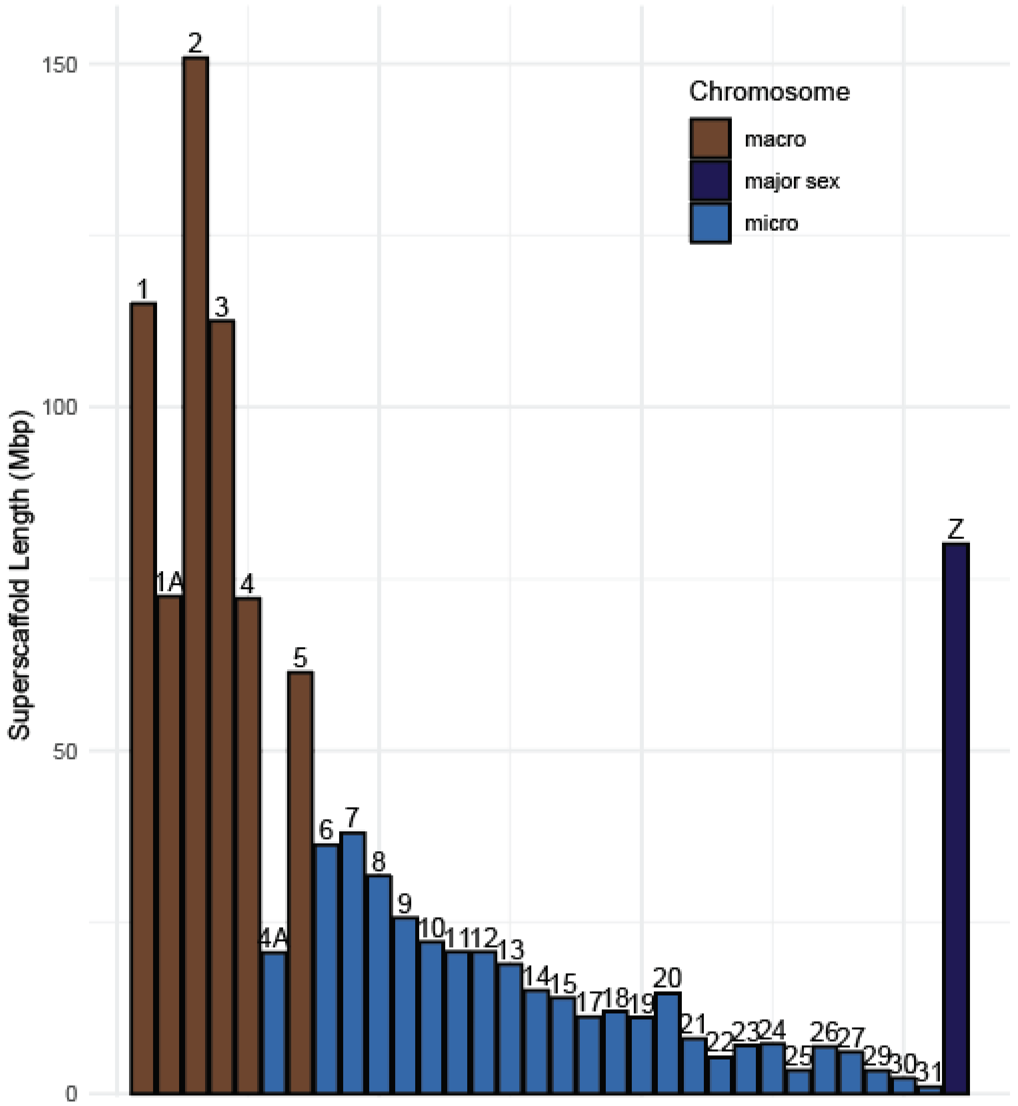

Figure S7 | pseudo-chromosome sizes with macro and micro chromosome numbers, based on zebra finch synteny, labelled.

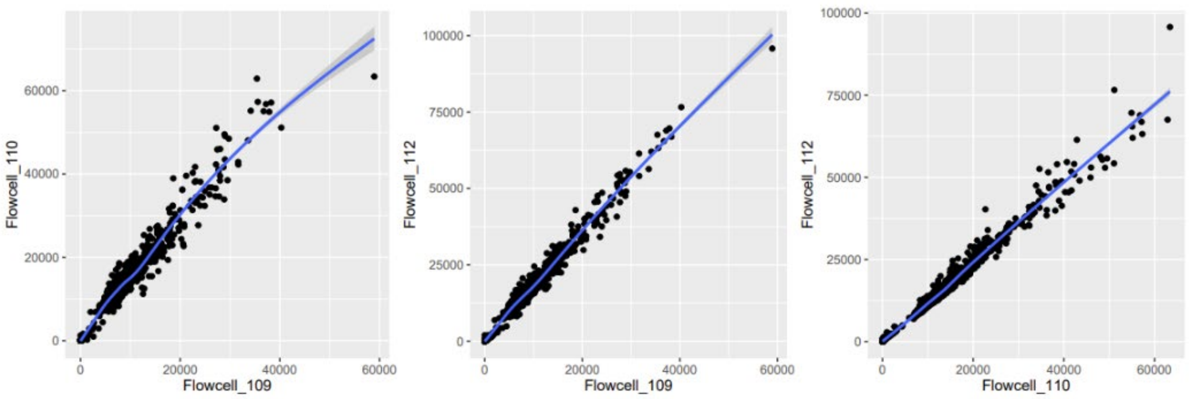

Figure S8 | Correlation plots of the methylation calls across the three flow cells obtained by linear regression in R.

Table S3 | Ensembl reference species used in the GEMOMA genome annotation.

Common Name	Scientifiec Name	Ensembl Assembly
Eurasian sparrowhawk	*Accipiter nisus*	Accipiter_nisus_ver1.0
Yellow-billed parrot	*Amazon collaria*	ASM394721v1
Mallard	*Anas platyrhynchos*	ASM874695v1
Pink-footed goose	*Anser brachyrhynchus*	ASM259213v1
Swan goose	*Anser cygnoides*	GooseV1.0
Greater spotted kiwi	*Apteryx haastii*	aptHaa1
Little spotted kiwi	*Apteryx owenii*	aptOwe1
Okarito brown kiwi	*Apteryx rowi*	aptRow1
Golden eagle	*Aquila chrysaetos chrysaetos*	bAquChr1.2
Burrowing owl	*Athene cunicularia*	athCun1
Small tree finch	*Camarhynchus parvulus*	STF_HiC
Golden pheasant	*Chrysolophus pictus*	Chrysolophus_pictus_GenomeV1.0
Blue tit	*Cyanistes caeruleus*	cyaCae2
Emu	*Dromaius novaehollandiae*	droNov1
Gouldian finch	*Erythrura gouldiae*	GouldianFinch
Flycatcher	*Ficedula albicollis*	FicAlb_1.4
Chicken	*Gallus gallus*	GRCg6a
Median ground-finch	*Geospiza fortis*	GeoFor_1.0
Bengalese finch	*Lonchura striata domestica*	LonStrDom1
Turkey	*Meleagris gallopavo*	Turkey_2.01
Great tit	*Parus major*	Parus_major1.1
Ring-necked pheasant	*Phasianus colchicus*	ASM414374v1
Common canary	*Serinus canaria*	SCA1
African ostrich	*Struthio camelus australis*	ASM69896v1
Zebra finch	*Taeniopygia guttata*	bTaeGut1_v1.p
White-throated sparrow	*Zonotrichia albicollis*	Zonotrichia_albicollis-1.0.1

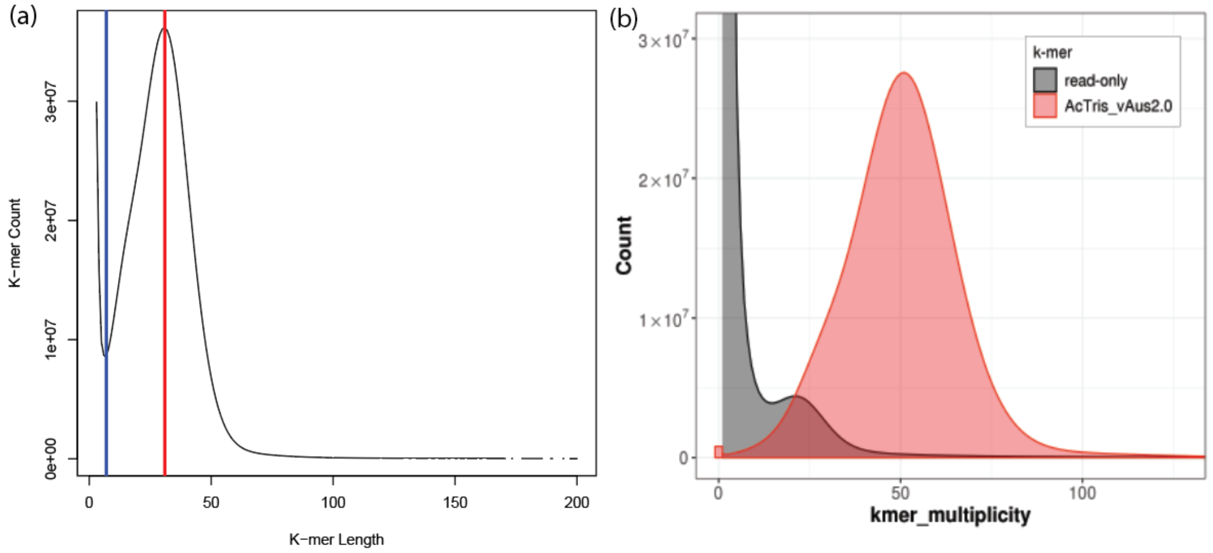

Figure S9 | Histogram of *Acridotheres tristis* linked read gDNA k-mer counts. Panel (a) was estimated using JELLYFISH from k=7 to k=200. Vertical red line denotes histogram peak at k=31. Genome Length = 36,028,219,169 / 31 = 1,162,200,618 bp. The final genome assembly size was used to find the using the genome size k-mer estimate. 1,040,603,622 / 1,162,200,618 = 89.54% complete. Panel (b) was estimated by MERQURY and reported a genome completeness score of 91.17% and a consensus quality value (QV) of 44.1.

Table S4 | Genome size and completion statistics produced by JELLYFISH for a range of initial k-mer values. Completion statistic based on the final AcTris_vAus2.0 assembly size of 1,040,603,622 bp.

**K-mer Value**	**18**	**19**	**20**	**21**	**22**
**Predicted Genome Size**	1,142,465,245	1,150,752,569	1,162,200,618	1,177,818,515	1,300,046,659
**Completion %**	91.08	90.43	89.54	88.35	80.04

Table S5 | Genome assembly statistics for the VGP *Acridotheres tristis* genome assembly (GCA_027559615.1) for comparison.

Assembly Statistic	VGP *Acridotheres tristis*	*AcTris_vAus2.0*
Total length (bp)	1,193,419,045	1,040,539,946
Number of scaffolds	455	256
Scaffold N50 (bp)	75,254,055	72,486,765
Scaffold L50	6	5
Largest scaffold (bp)	156,254,668	150,861,042
Mean scaffold length (bp)	2,622,899.00	4,064,609.16
Median scaffold length (bp)	123,340	3,369
Number of Contigs	890	597
Contig N50 (bp)	7,308,823	10,406,399
Contig L50	44	30
Gap (N) length (bp)	2,422,523	34,958 (0.00%)
GC content (%)	42.32%	41.85%
BUSCO (genome: Aves)	8338	8,338
Complete	8,094 (97.1%)	8,108 (97.2%)
Complete (single copy)	8,060 (96.7%)	8,077 (96.9%)
Complete (duplicated)	34 (0.4%)	31 (0.4%)
Fragmented	46 (0.6%)	44 (0.5%)
Missing	198 (2.4%)	186 (2.2%)

Table S6 | BUSCO statistics for the *Acridotheres tristis* AcTris_vAus2.0 genome annotations using BRAKER3 and GEMOMA.

Assembly Statistic	AcTris_vAus2.0
BRAKER3	
BUSCO (transcriptome: Aves)	8338
Complete	7341
Complete (single copy)	5599
Complete (duplicated)	1742
Fragmented	64
Missing	933
GEMOMA*	
BUSCO (transcriptome: Aves)	8338
Complete	8150
Complete (single copy)	8122
Complete (duplicated)	28
Fragmented	54
Missing	134

* Filtered for longest transcript

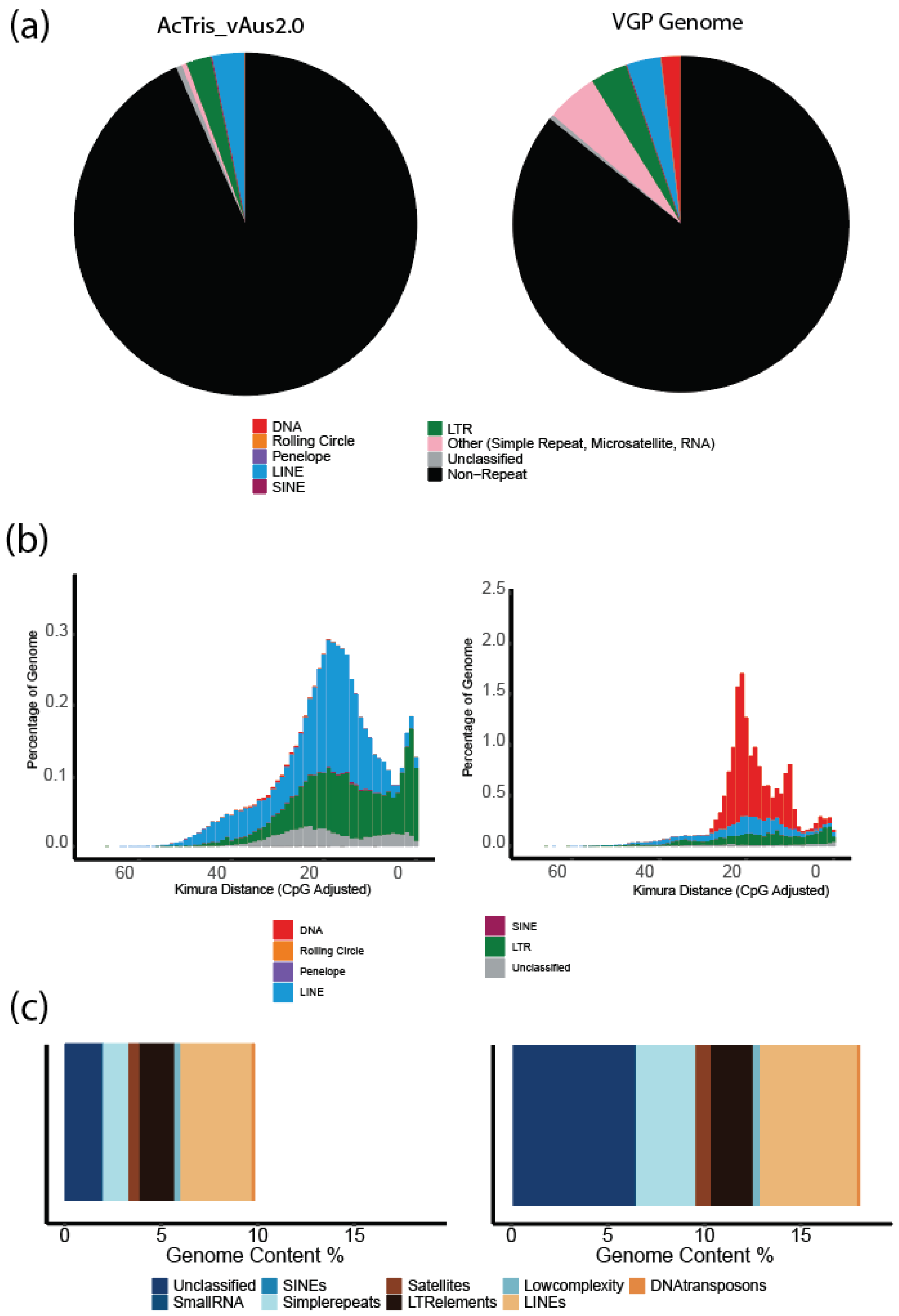

Figure S10 | Repeat content of the *Acridotheres tristis* AcTris_vAus2.0 genome (left) and the VGP A. tristis genome (right). Panels (a) and (b) are profiles of genome transposable element content as generated by EARLYGREY. Panel (c) is a REPEATMASKER repeat annotation of the two genome assemblies.

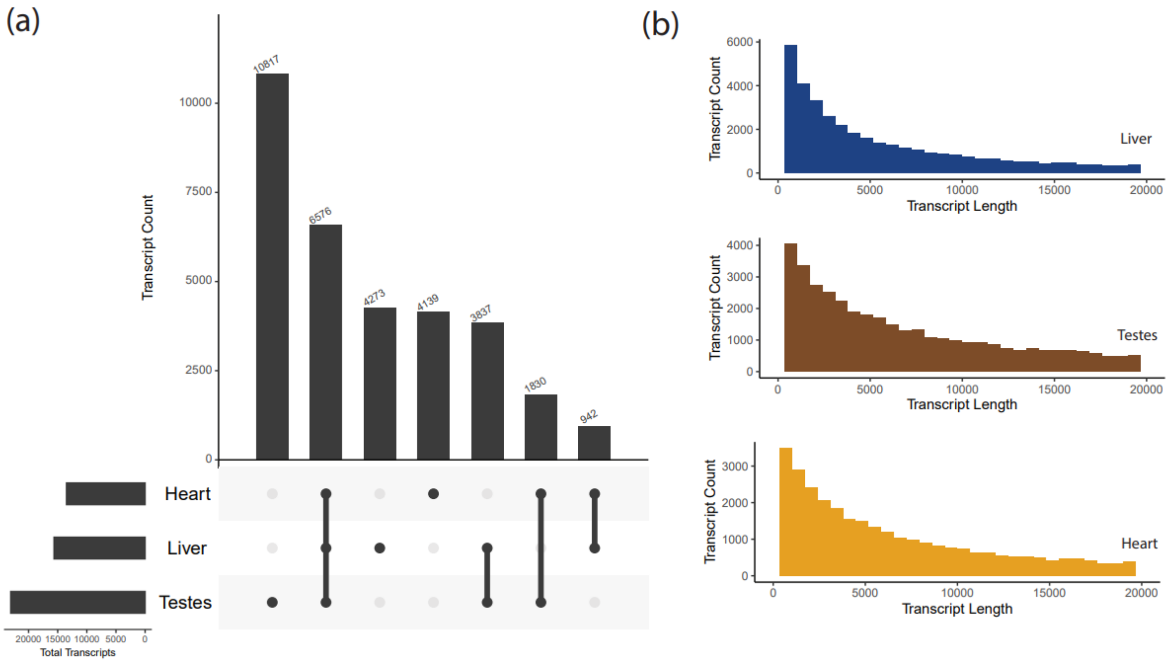

Figure S11 | ***Acridotheres tristis*** **transcriptome summary information**. Panel **(a)** depicts overlap between tissue-specific transcripts assembled by STRINGTIE v2.2.20, calculated using GFFCOMPARE v0.12.6 and visualised in R using the package UPSETR. Panel **(b)** depicts size classes of transcripts from each tissue.

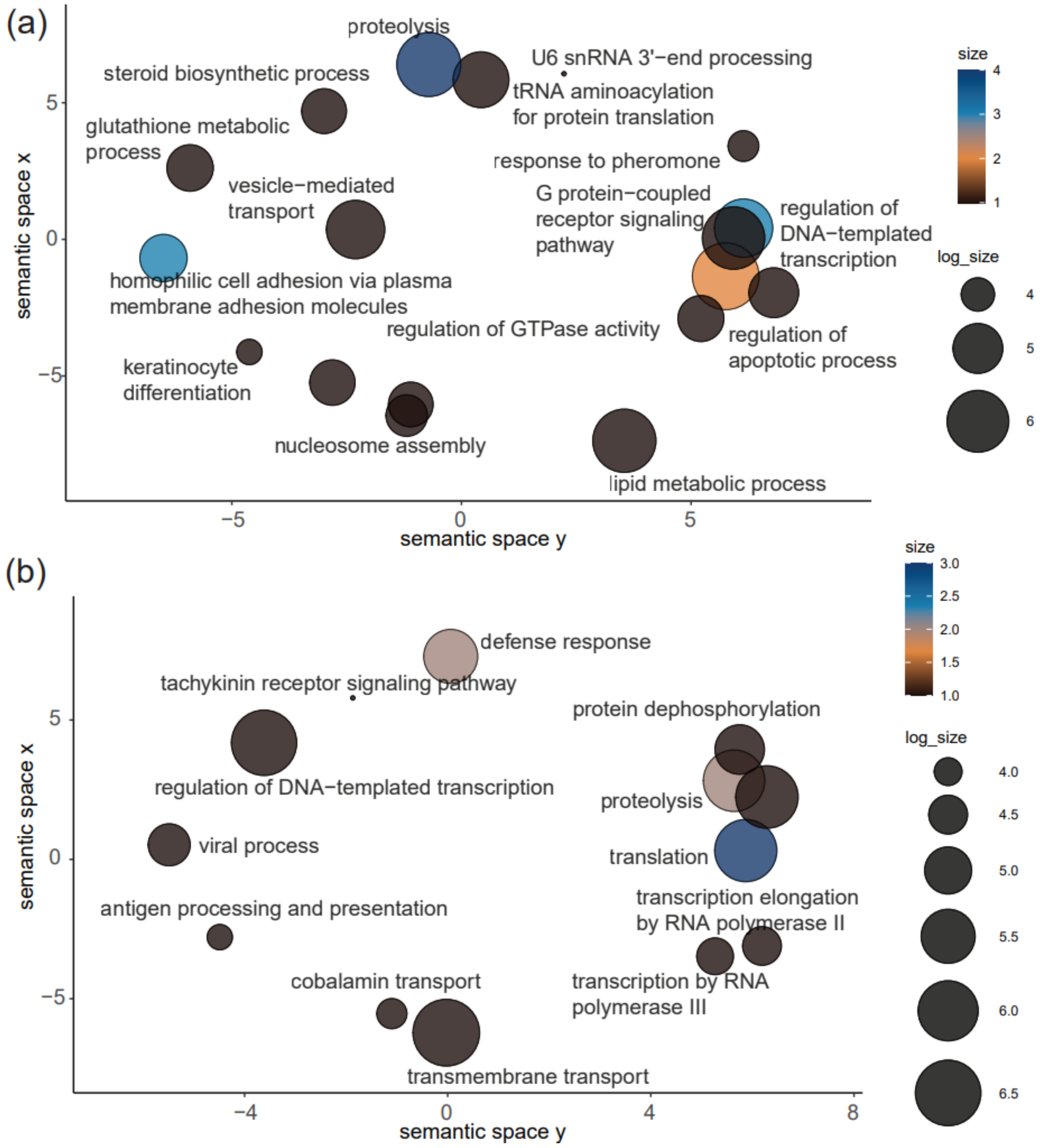

Figure S12 | REVIGO summary of the biological process captured in gene ontology terms associated with orthogroups determined to be to be under panel (a) significant expansion and panel (b) significant contractions in *A. tristis.* Colour represents across how many separate significantly expanded or contracted orthogroups the GO term was independently annotated with using INTERPROSCAN, while log size represents the weighting of that GO term in the dataset once redundant GO terms were collapsed during REVIGO analysis and visualisation. Therefore, both colour and circle size capture the level of representation of the term but do so at different steps of the analysis.

Table S8 | Summary of GO terms reported on Figure S12, and whether they appeared in the list of expanded or contracted HOGs.

GO Term	Name	Event
GO:0006334	nucleosome assembly	Expansion
GO:0006355	regulation of DNA-templated transcription	Expansion
GO:0006418	tRNA aminoacylation for protein translation	Expansion
GO:0006508	proteolysis	Expansion
GO:0006629	lipid metabolic process	Expansion
GO:0006694	steroid biosynthetic process	Expansion
GO:0006749	glutathione metabolic process	Expansion
GO:0007156	homophilic cell adhesion via plasma membrane adhesion molecules	Expansion
GO:0007186	G protein-coupled receptor signaling pathway	Expansion
GO:0016192	vesicle-mediated transport	Expansion
GO:0019236	response to pheromone	Expansion
GO:0030216	keratinocyte differentiation	Expansion
GO:0031424	keratinization	Expansion
GO:0031175	neuron projection development	Expansion
GO:0034477	U6 snRNA 3'-end processing	Expansion
GO:0035556	intracellular signal transduction	Expansion
GO:0042981	regulation of apoptotic process	Expansion
GO:0043087	regulation of GTPase activity	Expansion
GO:0051260	protein homooligomerization	Expansion
GO:0006355	regulation of DNA-templated transcription	Contraction
GO:0006368	transcription elongation by RNA polymerase II	Contraction
GO:0006383	transcription by RNA polymerase III	Contraction
GO:0006412	translation	Contraction
GO:0006468	protein phosphorylation	Contraction
GO:0006470	protein dephosphorylation	Contraction
GO:0006508	proteolysis	Contraction
GO:0006952	defense response	Contraction
GO:0007217	tachykinin receptor signaling pathway	Contraction
GO:0015889	cobalamin transport	Contraction
GO:0016032	viral process	Contraction
GO:0019882	antigen processing and presentation	Contraction
GO:0006955	immune response	Contraction
GO:0045087	innate immune response	Contraction
GO:0055085	transmembrane transport	Contraction

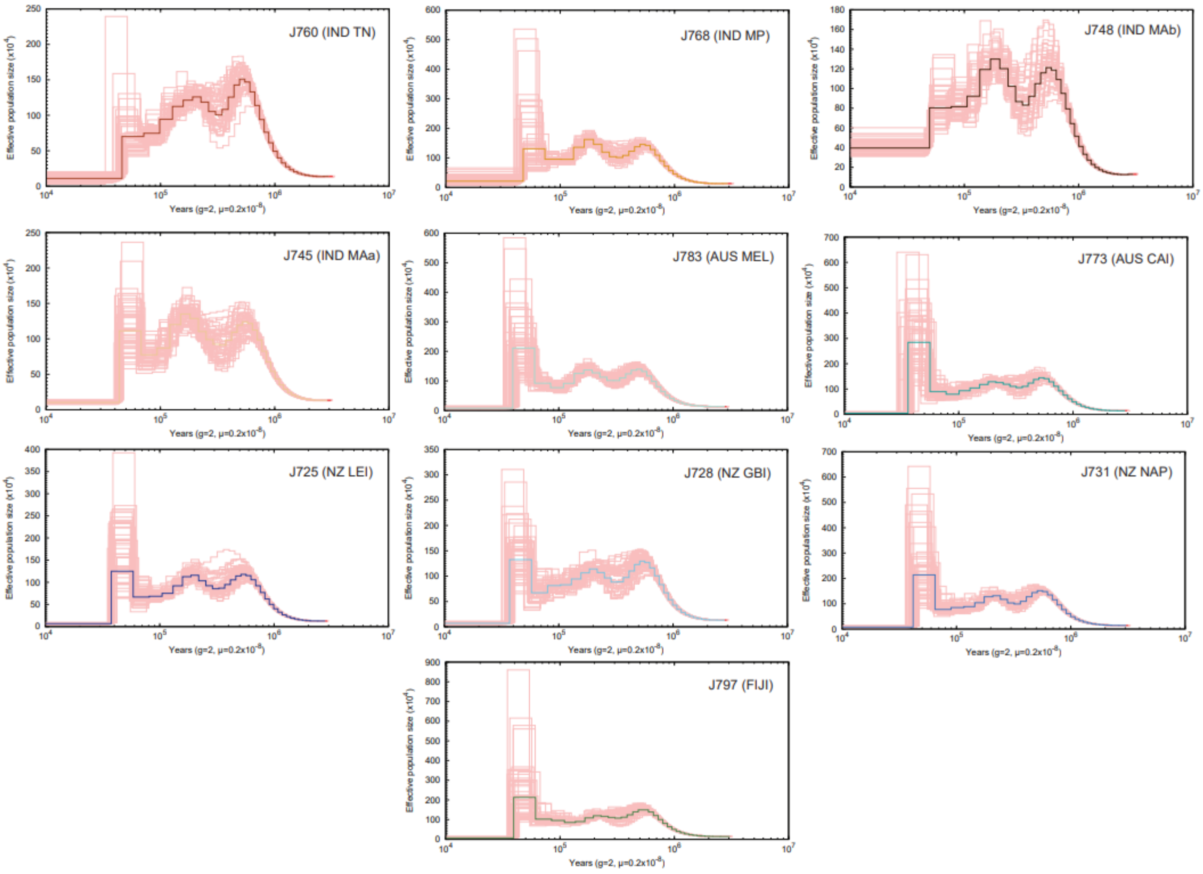

Figure S13 | PSMC bootstrapping plots with colour scheme matching Fig. 6 from the main manuscript indicated next to each plot.

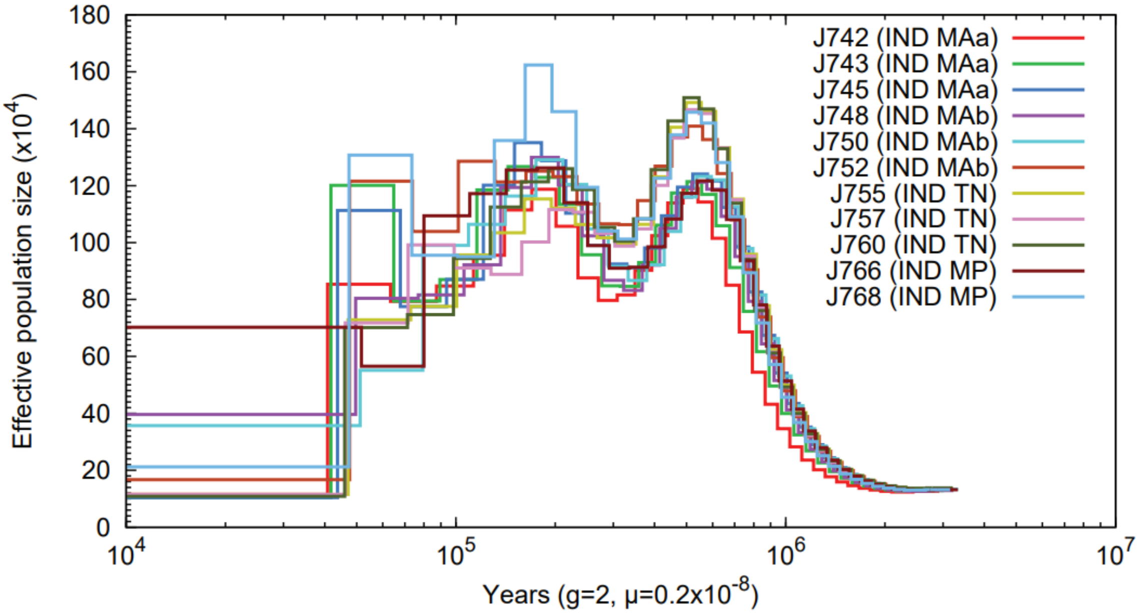

Figure S14 | PSMC plots for native range individuals.
